# State feedback control design for Boolean networks

**DOI:** 10.1186/s12918-016-0314-z

**Published:** 2016-08-26

**Authors:** Rongjie Liu, Chunjiang Qian, Shuqian Liu, Yu-Fang Jin

**Affiliations:** 1 Department of Electrical and Computer Engineering, The University of Texas at San Antonio, San Antonio, 78249 TX United States; 2 Department of Biomedical Engineering, Northwestern University, Evanston, IL USA; 3 San Antonio Cardiovascular Proteomics Center, San Antonio, Texas USA

**Keywords:** Boolean network, State feedback control, Controllability

## Abstract

**Background:**

Driving Boolean networks to desired states is of paramount significance toward our ultimate goal of controlling the progression of biological pathways and regulatory networks. Despite recent computational development of controllability of general complex networks and structural controllability of Boolean networks, there is still a lack of bridging the mathematical condition on controllability to real boolean operations in a network. Further, no realtime control strategy has been proposed to drive a Boolean network.

**Results:**

In this study, we applied semi-tensor product to represent boolean functions in a network and explored controllability of a boolean network based on the transition matrix and time transition diagram. We determined the necessary and sufficient condition for a controllable Boolean network and mapped this requirement in transition matrix to real boolean functions and structure property of a network. An efficient tool is offered to assess controllability of an arbitrary Boolean network and to determine all reachable and non-reachable states. We found six simplest forms of controllable 2-node Boolean networks and explored the consistency of transition matrices while extending these six forms to controllable networks with more nodes. Importantly, we proposed the first state feedback control strategy to drive the network based on the status of all nodes in the network. Finally, we applied our reachability condition to the major switch of P53 pathway to predict the progression of the pathway and validate the prediction with published experimental results.

**Conclusions:**

This control strategy allowed us to apply realtime control to drive Boolean networks, which could not be achieved by the current control strategy for Boolean networks. Our results enabled a more comprehensive understanding of the evolution of Boolean networks and might be extended to output feedback control design.

## Background

Boolean networks have been successfully applied to model gene regulations and protein interactions for the last two decades because the up or down regulation of molecular expressions can be described as discrete Boolean functions [1–4]. In these applications, molecules and their interactions were treated as nodes and edges, respectively. Boolean networks were characterized with network structure, i.e. the organization of nodes and edges, and the interactive Boolean functions among the nodes [5]. Recent advances in high through-put technology such as genomics and proteomics have prompted us to determine the interactions among molecules, thus establishing a Boolean network for a small biological system is feasible.

Currently, the most common senorio of biological experiments is to modify a specific molecular expression through gene knock-out or dosage injection and to evaluate the down stream effects of the modified molecule by examining expressions of a panel of genes based on expertise knowledge or using unbiased screening. Such experimental design only changes the initial state of a biological network and no other stimuli (control input) is introduced to the system during the response. Further, such experimental design was performed without answering the following questions: 1) whether changing the state of one node or a group of nodes of a network will drive the network to desired states; and 2) how to determine the effect of structural and functional changes of a network.

Similar questions have been answered for linear time invariant systems as reachability and controllability of a system. In general, a particular state *x*_1_ is reachable if there exists a control input to transfer the system from any initial state to *x*_1_ in a finite time. Meanwhile, a system is defined as reachable if every state of the system is reachable [6]. Controllability of a system is very similar to reachability definition, which means if there exists a control input to transfer the system from any initial condition to the origin in finite time. For a linear time invariant system, we can always translate a state to the origin using coordinate transformation. And therefore, reachability is a fundamental check for controllability.

Preliminary results on controllability of general networks were obtained via pinning control strategy in terms of the spectral properties of network structure [7]. Barabasi’s group has mapped controllability conditions of linear time invariant systems to complex networks and computationally determined the driver nodes for a network [8]. Their results answered the question which nodes might affect the progression of a network. Yuan and colleagues further examined the effect of weights of the edges on controllability of a general network [9]. Both results focused on finding the minimal number of nodes to control the network. However, these results are computational analysis due to the lack of mathematical representation of complex networks. In the year of 2003, Cheng proposed a mathematical representation of Boolean networks with semi-tensor product [10], which provided a possible approach to systemically examine the controllability of Boolean Networks. Sun and Cheng defined the controllability of a Boolean network and obtained preliminary controllable condition on network structure [11–13]. However, the definition and conditions were mathematical oriented and have not been linked to Boolean operations in real networks, which imposed extra difficulty for users without the required mathematical background.

In this study, we defined both structural and functional requirements for a reachable Boolean network using semi-tensor product. We found 6 forms for controllable 2-node Boolean networks with both structural and functional conditions, developed a sharable tool to determine whether an arbitrary Boolean network is reachable or not, and gave possible structural and functional changes to modify the reachability. Most importantly, we proposed the first state feedback control strategy to drive a Boolean network by integrating current status of all nodes in the network. The control strategy allowed realtime application and will provide effective control to drive the network to a desired state.

### Boolean networks

Boolean networks proposed by Kauffman are discrete-time dynamics systems with Boolean state-variables [5]. Each node of a Boolean network is a Boolean state variable with logic value 0 (false) or 1 (true) corresponding to down or up regulation of a molecule in a biological network. States of all nodes in a Boolean network will lead to a Boolean vector.

A Boolean function with *k* variables is a mapping B: {0,1}^*k*^→{0,1} from the set of all *k*-tuples over {0,1} to a binary output. This function describes how to determine a Boolean-valued output based on certain logical operations from *k* binary inputs. It can also be interpreted as how the expression of a molecule will be determined by other *k* molecules interacting with it. The basic Boolean operations include AND (conjunction), OR (union), and NOT (inhibition). A list of sixteen logical operations was shown in Table 1.

**Table 1:** Logical matrices of 16 Boolean operations

Logical connective	Logical operator	Logical matrix for 2-node
	symbol	Boolean networks
True	⊤	$\left (\begin {array}{cccc} 1&1&1&1 \\ 0&0&0&0 \end {array} \right)$
False	⊥	$\left (\begin {array}{cccc} 0&0&0&0 \\ 1&1&1&1 \end {array} \right)$
Proposition *x*_1_	*x*_1_	$\left (\begin {array}{cccc} 1&1&0&0 \\ 0&0&1&1 \end {array} \right)$
Proposition *x*_2_	*x*_2_	$\left (\begin {array}{cccc} 1&0&1&0 \\ 0&1&0&1 \end {array} \right)$
Negation (inhibition) *x*_1_	¬*x*_1_	$\left (\begin {array}{cccc} 0&0&1&1 \\ 1&1&0&0 \end {array} \right)$
Negation (inhibition) *x*_2_	¬*x*_2_	$\left (\begin {array}{cccc} 0&1&0&1 \\ 1&0&1&0 \end {array} \right)$
Conjunction	∧	$\left (\begin {array}{cccc} 1&0&0&0 \\ 0&1&1&1 \end {array} \right)$
Disjunction (union)	∨	$\left (\begin {array}{cccc} 1&1&1&0 \\ 0&0&0&1 \end {array} \right)$
Converse implication	←	$\left (\begin {array}{cccc} 1&1&0&1 \\ 0&0&1&0 \end {array} \right)$
Material conditional	→	$\left (\begin {array}{cccc} 1&0&1&1 \\ 0&1&0&0 \end {array} \right)$
Converse nonimplication	$\nleftarrow $	$\left (\begin {array}{cccc} 0&0&1&0 \\ 1&1&0&1 \end {array} \right)$
Material nonimplication	$\nrightarrow $	$\left (\begin {array}{cccc} 0&1&0&0 \\ 1&0&1&1 \end {array} \right)$
Biconditional	⇔	$\left (\begin {array}{cccc} 1&0&0&1 \\ 0&1&1&0 \end {array} \right)$
Alternative denial	*↑*	$\left (\begin {array}{cccc} 0&1&1&1 \\ 1&0&0&0 \end {array} \right)$
Joint denial	*↓*	$\left (\begin {array}{cccc} 0&0&0&1 \\ 1&1&1&0 \end {array} \right)$
Exclusive disjunction	⊕	$\left (\begin {array}{cccc} 0&1&1&0 \\ 1&0&0&1 \end {array} \right)$

### Algebraic representation of Boolean networks

A Boolean network with *n* logical variables *V*_*i*_,*i*=1,2,…,*n* and *m* control inputs *u*_*j*_,*j*=1,2,…,*m* can be expressed as

1 $$\begin{array}{@{}rcl@{}} V_{1}(t+1)&=&B_{1}(V_{1}(t),\ldots,V_{n}(t),u_{1}(t),\ldots,u_{m}(t))\\ &\vdots&\\ V_{n}(t+1)&=&B_{n}(V_{1}(t),\ldots,V_{n}(t),u_{1}(t),\ldots,u_{m}(t)), \end{array} $$

where *V*_*i*_ and *u*_*j*_ take value from the set {0,1} [14]. The representation of each Boolean function is defined as *B*_*i*_:{0,1}^*n*+*m*^→{0,1},*i*=1,…,*n*, which is preassigned Boolean logical functions determined by the biological process. For a n-node boolean network, there are 2^*n*^ possible states. If there is no control input *u*_*j*_, *B*_*i*_ is a 2×2^*n*^ matrix because each logical value 0 or 1 is expressed as a vector (0,1)^*T*^ or (1,0)^*T*^, respectively. The algebraic state-space representation of the Boolean control network is set up based on the semi-tensor product of matrices which will be introduced in our method part [10, 14, 15].

For each Boolean function, there is a unique truth table while the algebraic expression of a Boolean function is not unique. This means that there exist different forms of structures and operations of a network with same Boolean function. In this study, we assume each Boolean function is represented with the simplest form to reduce the complexity of analysis.

## Results

We first defined all reachable states of a Boolean network with control applied at the beginning and then removed the control input from the system. This exactly mimics the situation of modifying one node or a group of nodes in the network initially and examining the response. We then extended the reachability to controllability.

### Determining reachability using graphical approach

For a n-node Boolean network, an integrated state represents the status of *n* variables in the network. All together there are 2^*n*^ integrated states, representing each possible status of the *n* nodes. An integrated state is denoted as $e_{2^{n}}^{j}$, *j*=1,2,⋯,2^*n*^, in which $e_{2^{n}}^{j}$ means the *j*_*th*_ column of 2^*n*^×2^*n*^ identity matrix. A graphical representation, time transition diagram, was proposed to illustrate the transition among the integrated states. Each **node** of the time transition diagram corresponds to one integrated state $e_{2^{n}}^{j}$ of a dynamic network. A directed edge from $e_{2^{n}}^{j}$ to $e_{2^{n}}^{k}$, *j*,*k*=1,2,⋯,2^*n*^, indicates temporal transitions from an integrated state $e_{2^{n}}^{j}$ to an integrated state $e_{2^{n}}^{k}$. The directed edge also represents that the *j*_*th*_ column in the transition matrix is $e_{2^{n}}^{k}$. The transition matrix of a Boolean network is calculated using semi-tensor product, and each column of the transition matrix is a vector $e_{2^{n}}^{k}$. From the left to the right, each column of the transition matrix represents the transition from $e_{2^{n}}^{j}$, *j* increasing from 1 to 2^*n*^, to its next integrated state represented by a column vector $e_{2^{n}}^{k}$. Specifically, the left most column of the transition matrix represents the transition from $e_{2^{n}}^{1}$ to its next integrated state, and the right most column in the matrix represents the transition from $e_{2^{n}}^{2^{n}}$ to its next integrated state. Therefore, there are a total of 2^*n*^ outgoing arrows in the time transition diagram and a node may have multiple incoming arrows but has only one outgoing arrow.

Reachability of a node in the time transition diagram means the corresponding integrated state can be reached from any initial integrated state in finite time. If each node in the time transition diagram is reachable, the Boolean network is reachable.

#### **Finding 1**

A Boolean network with *n* nodes (*n*>1) is reachable if and only if the signal flow goes through each node in the time transition diagram by one direction, indicating that each node has one outgoing arrow and one incoming arrow.

There are some specific properties for the transition matrix of a reachable Boolean network: 1) There is only one 1 in each column and each row, suggesting an integrate state can only be reached by one other integrated state; 2) Every diagonal elements is zero. It means that the *j*_*th*_ column is not $e_{2^{n}}^{j}$. This property excludes self transition of one integrated state. 3) If the *j*_*th*_ column is $e_{2^{n}}^{k}$, then the *k*_*th*_ column is not $e_{2^{n}}^{j}$, *n*≥2, which excludes transition between two integrated states. However, this property is not true for a 1-node reachable Boolean network. The transition matrix of 1-node reachable boolean network satisfies that the 1_*st*_ column is ${e_{2}^{2}}$ while the 2_*nd*_ column is ${e_{2}^{1}}$.

**Fig. 1:**
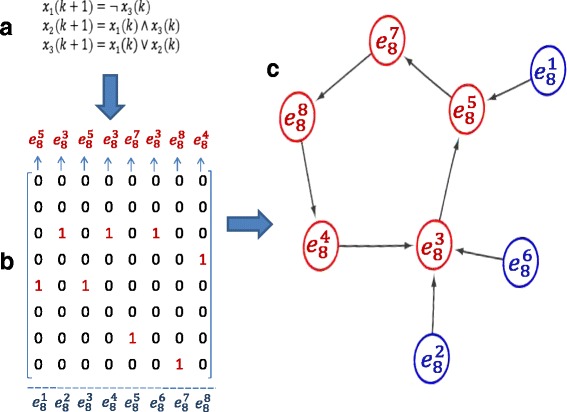
Determination of the reachability of a three-node Boolean network with given Boolean functions. Based on the logical operations (**a**) for this network, the corresponding time transition matrix (**b**) and the time transition diagram (**c**) can be obtained by semi-tensor product. A signal flow among five integrated states ${e_{8}^{3}}\to {e_{8}^{5}}\to {e_{8}^{7}}\to {e_{8}^{8}}\to {e_{8}^{4}}\to {e_{8}^{3}}$ is formed as a circle. According to Finding 1, it means that all these five integrated states are reachable, which are highlighted in *red*, while the other three states ${e_{8}^{1}}$, ${e_{8}^{2}}$ and ${e_{8}^{6}}$ are not reachable, which are highlighted in *blue*

Here, an example of a 3-node Boolean network is presented in Fig. 1 to show how the reachability is determined and all 8 integrated states representing possible status of the 3 nodes in the Boolean network are listed in Table 2. Based on these integrated states listed in Table 2 and time transition diagram in Fig. 1, whatever changes we make to the nodes through knock out of a node (value 0) nor dosage injection to a node (value 1), the network can not reach the integrated state ${e_{8}^{1}}$ (node 1 is 0, node 2 is 1, and node 3 is 1), ${e_{8}^{2}}$ (node 1 is 1, node 2 is 1, and node 3 is 0), ${e_{8}^{6}}$ (node 1 is 0, node 2 is 1, and node 3 is 0). If we force the initial status of the system to be these three states, the network will deviate from these states and never come back. This result can provide a guideline for experiment design to examine down stream effect for a giving pathway with known Boolean network. For the network shown in Fig. 1, when ${e_{8}^{1}}$, or ${e_{8}^{2}}$, or ${e_{8}^{6}}$ is a desired state we would like the network to go, a more complicated control strategy should be introduced in stead of just modify status of one node of a group of nodes.

**Table 2:** Relationship between eight integrated states of a 3-node Boolean network and logical values of the 3 nodes

Node 1	Node 2	Node 3	Integrated state
1	1	1	${e_{8}^{1}}$
1	1	0	${e_{8}^{2}}$
1	0	1	${e_{8}^{3}}$
1	0	0	${e_{8}^{4}}$
0	1	1	${e_{8}^{5}}$
0	1	0	${e_{8}^{6}}$
0	0	1	${e_{8}^{7}}$
0	0	0	${e_{8}^{8}}$

**Fig. 2:**
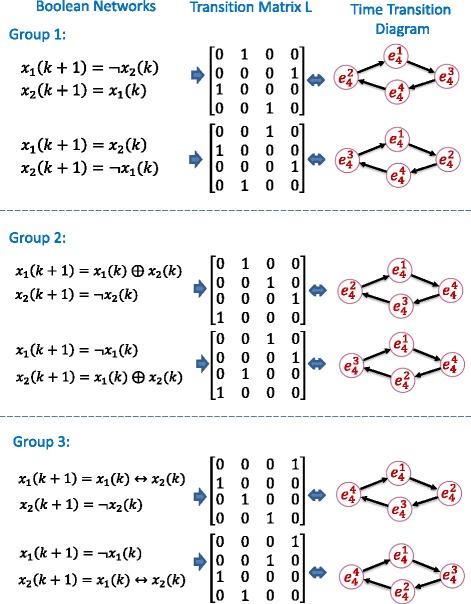
The six simplest 2-node reachable Boolean networks with their logic operations. The left column shows simplest reachable Boolean functions of two variables, the middle column represents the state transition matrix, and the right column illustrates the time transition diagram among four integrated states of two variables. The four integrated states of all six Boolean networks are all reachable

**Reachable 2-node Boolean network with logical operations.** We examined all 2-node Boolean networks with combinations of 16 logical operations as shown in Table 1. We found that there were only six simplest forms of reachable 2-node Boolean networks. These six Boolean networks were shown in Fig. 2 with their corresponding time transition diagrams and transition matrices.

Interestingly, these six simplest networks show highly coupled property, which can be divided into three groups. In each group, if state *x*_1_ is swapped with *x*_2_ in one of the coupled networks, it exactly becomes the other network. Therefore, for any given 2-node Boolean network dynamics with logical operations, it will be straightforward to know that it is reachable or not when it reduces to its simplest form. In addition, this provided a baseline to check reachability and controllability of a Boolean network with more nodes.

**Feedback control design for N-node lower-triangle Boolean networks** Starting from the known 6 forms of 2-node reachable Boolean networks, their extensions to N-node Boolean networks can be derived based on the property of transition matrix. Further, for the extended N-node Boolean network with control input added to the *nth* node directly, the feedback control input can be designed to implement the reachability of the N-node Boolean network.

#### **Finding 2**

For a given N-node lower-triangle Boolean network dynamic with control input located at the *nth* node, if the first N-1 Boolean network dynamic is a reachable (N-1)-node Boolean dynamics, a feedback control can be designed, which is extracted from the *N*_*th*_ logical function of extended N-node reachable Boolean dynamics from the (N-1)-node reachable Boolean dynamics.

Given one of the 6 reachable 2-node boolean networks in Fig. 2, we can extend the network with extra nodes once the added boolean functions guarantee the time transition diagram satisfy the condition in our 1st finding. For an extended N-node reachable Boolean network, if we divide its (2^*n*^×2^*n*^ transition matrix *L*_*N*_ into sub-blocks, and define 0-block as a square matrix with all zero elements, and 1-block as square matrix with non-zero element, the structure of the transition matrix *L*_*N*_ in terms of the sub-blocks will mimic the transition matrix for boolean networks with less nodes.

**Fig. 3:**
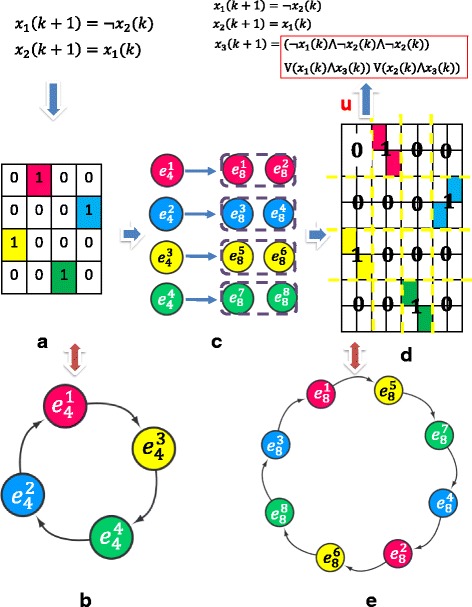
The pipeline of extended 3-node reachable Boolean network from 2-node reachable Boolean network. If transition matrix *L*_3_(2^3^×2^3^) of 3-node Boolean network system, is divided into 4×4 blocks, then the new transition matrix represented by the 4×4 matrix is exactly the same as transition matrix *L*_2_ of fundamental 2-node Boolean network dynamic. **a** The transition matrix of a 2-node reachable network; (**b**) Time transition diagram of 2-node network; (**c**) Each 1-block is extended to two 1-blocks; (**d**) The transition matrix of extended 3-node extended reachable network; (**e**) Corresponding time transition diagram of extended 3-node extended network

Specifically, if 1-block in transition matrix of 2-node network appears at row *i* and column *j*, then for a 3-node network extended from 2-node network, the two 1-blocks only appear at row 2*i*−1 and column 2*j*−1, row 2*i* and column 2*j* or at row 2*i*−1 and column 2*j*, row 2*i* and column 2*j*−1 respectively. An example of how to design the feedback control input of the 3-node Boolean network is shown below, which extends from 2-node reachable Boolean network. And the relationship between transition matrices was shown in Fig. 3. Further, the Boolean function for the 3rd node can be treated as control input *u* as shown below,

$$ \begin{aligned} x_{1}(k+1)=&\neg x_{2}(k)\\ x_{2}(k+1)=&x_{1}(k)\\ x_{3}(k+1)=&u,  \end{aligned}  $$

where u is the control input of the lower-triangle dynamic, which will be designed later.

For the 2-node reachable Boolean network represented by

$$ \begin{aligned} x_{1}(k+1)=&\neg x_{2}(k)\\ x_{2}(k+1)=&x_{1}(k), \end{aligned}  $$

we illustrate the inter relationship between the transition matrices and time transition diagram. Based on one possible transition matrix that guarantees the reachability of each integrated state, the boolean operation matrix M can be obtained and the corresponding boolean function for the 3rd node is determined. With the possible transition matrix shown in Fig. 3, the corresponding Boolean function is listed as

$$ \begin{aligned} &{}x_{1}(k+1)=\neg x_{2}(k)\\ &{}x_{2}(k+1)=x_{1}(k)\\ &{}x_{3}(k+1)=(\neg x_{1}(k)\wedge\neg x_{2}(k)\wedge\neg x_{3}(k))\\ &\times\vee(x_{1}(k)\wedge x_{3}(k))\vee(x_{2}(k)\wedge x_{3}(k)). \end{aligned}  $$

Then, the feedback control input u is designed as

$$ {{\begin{aligned} {}u\,=\,(\neg x_{1}(k)\!\wedge\!\neg x_{2}(k)\wedge\neg x_{3}(k))\!\vee(x_{1}(k)\wedge x_{3}(k))\vee\!(x_{2}(k)\wedge x_{3}(k)). \end{aligned}}}  $$

### Analysis of reachability for P53 pathway

**Fig. 4:**
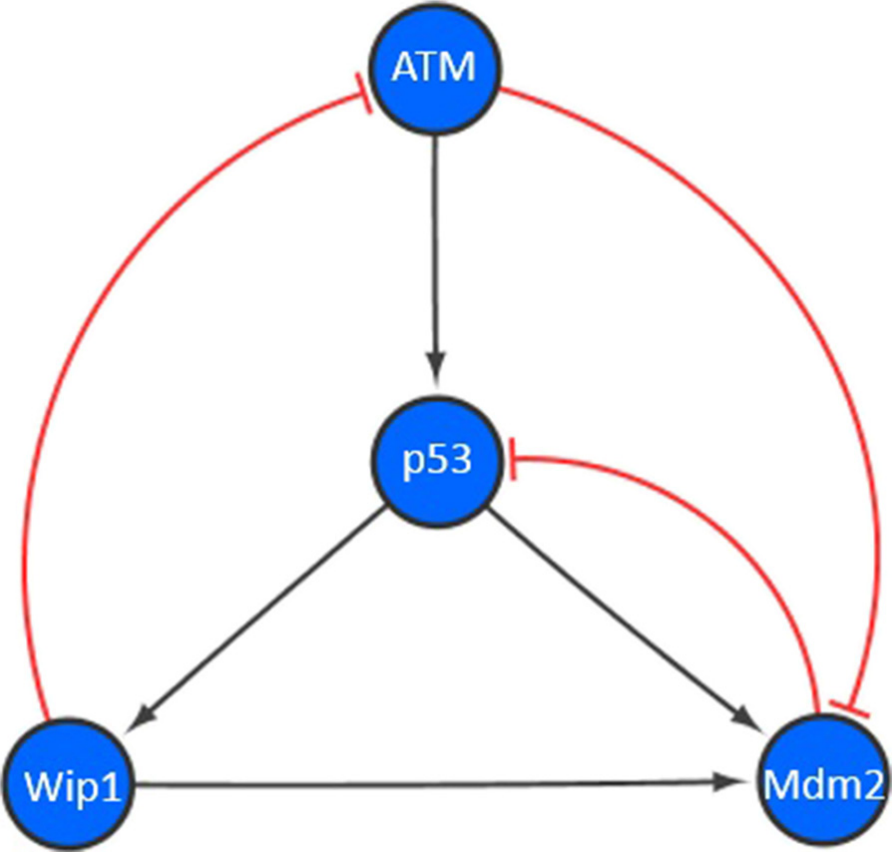
The major switch of p53 pathway. The major interactions for p53 pathway, were presented among four nodes: ‘ATM’, ‘p53’, ‘Wip1’, ‘Mdm2’ respectively. The red line means the inhibition impact while the *black line*stands for the promotion impact

The p53 pathway responds to intrinc and extrinsic stress signals that can disrupt the fidelity of DNA replication, genome stability, cell cycle progression, and cell division. The pathway contains complicated feedback regulatory mechanisms and many experimental results have been accumulated to illustrate the regulations. In the major switch of p53 pathways as shown in Fig. 4, there are four state nodes are denoted as *x*_1_, *x*_2_, *x*_3_ and *x*_4_, which present as ‘ATM’, ‘p53’, ‘Wip1’, ‘Mdm2’, respectively [16]. The relationship between integrated states and its corresponding Boolean values of four genes is shown in Table 3 below.

**Table 3:** The relationship between integrated states and its corresponding Boolean values of four genes

ATM	p53	Wip1	Mdm2	Integrated state
1	1	1	1	$e_{16}^{1}$
1	1	1	0	$e_{16}^{2}$
1	1	0	1	$e_{16}^{3}$
1	1	0	0	$e_{16}^{4}$
1	0	1	1	$e_{16}^{5}$
1	0	1	0	$e_{16}^{6}$
1	0	0	1	$e_{16}^{7}$
1	0	0	0	$e_{16}^{8}$
0	1	1	1	$e_{16}^{9}$
0	1	1	0	$e_{16}^{10}$
0	1	0	1	$e_{16}^{11}$
0	1	0	0	$e_{16}^{12}$
0	0	1	1	$e_{16}^{13}$
0	0	1	0	$e_{16}^{14}$
0	0	0	1	$e_{16}^{15}$
0	0	0	0	$e_{16}^{16}$

The Boolean network representation of 4 genes is

2 $$ \begin{aligned} x_{1}(k+1)=&\neg x_{3}(k)\\ x_{2}(k+1)=& x_{1}(k)\wedge (\neg x_{4}(k))\\ x_{3}(k+1)=&x_{2}(k)\\ x_{4}(k+1)=&\neg x_{1}(k)\wedge (x_{2}(k)\vee x_{3}(k)) \end{aligned}  $$

The transition matrix is

3 $$ {{\begin{aligned} {}L\,=\,\left(e_{16}^{14}, e_{16}^{10}, e_{16}^{6}, e_{16}^{2}, e_{16}^{16}, e_{16}^{12}, e_{16}^{8}, e_{16}^{4}, e_{16}^{13}, e_{16}^{13}, e_{16}^{5}, e_{16}^{5}, e_{16}^{15}, e_{16}^{15}, e_{16}^{8}, e_{16}^{8}\right) \end{aligned}}}  $$

**Fig. 5:**
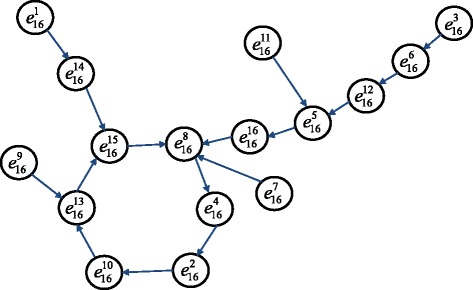
The time transition diagram of sixteen integrated states of 4 nodes in p53 pathway. The *solid lines* present the time path. As time goes on, any initial integrated state will reach a signal flow including six integrated states $e_{16}^{8}\to e_{16}^{4}\to e_{16}^{2}\to e_{16}^{10}\to e_{16}^{13}\to e_{16}^{15}\to e_{16}^{8}$. This phenomena induces that the states change periodically after a period of time

The corresponding time transition diagram is shown in Fig. 5. From the time transition diagram, there exists a cycle including ${e_{16}^{8}, e_{16}^{4}, e_{16}^{2}, e_{16}^{10}, e_{16}^{13}, e_{16}^{15}}$, suggesting a stable pulse generated by P53 pathway switches. Based on Table 3, each integrated state corresponds the specific values of four states. In Fig. 5, the high expression level of a gene presents Boolean value ‘1’ while low expression level means Boolean value ‘0’.

**Fig. 6:**
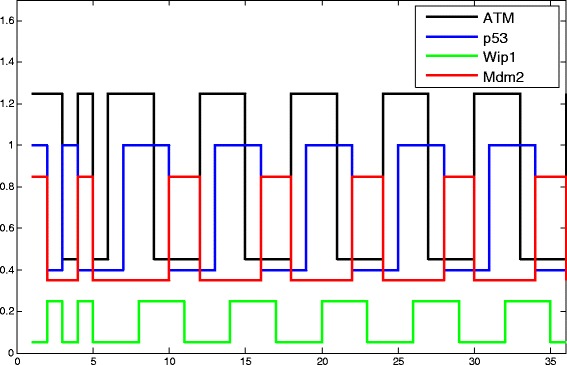
The pulses of p53 pathway. Expression levels of four genes in the major switch of P53 pathway lead to pulse diagram. The high expression level of a gene presents Boolean value ‘1’ while low expression level means Boolean value ‘0’. Expression levels of each node also lead to a specific integrated state in the time transition diagram. The four different pulse lines, which are ATM (*black solid line*), p53 (*blue solid line*), Wip1 (*green solid line*), Mdm2 (*red solid line*), show cyclic changes after 10 sec

Additionally, this stable pulse can be reached by different initial integrated states. One of the time course, which includes the main loop, is presented in Fig. 6 based on our simulation. The network exhibits the one-phase or two-phase dynamic, which depends on the initial states. If the initial is one of $e_{16}^{8}, e_{16}^{4}, e_{16}^{2}, e_{16}^{10}, e_{16}^{13}, e_{16}^{15}$, there exists only one-phase pulse, i.e. steady state pulse, which is a periodical pulse. If the initial states are others integrated states, there exists the two-phase pulse (transient pulse and steady state pulse), where the first phase is depends on the time distance between any state belongs to the periodical circle and the initial states and it ends at reaching any one state in the $e_{16}^{8}\to e_{16}^{4}\to e_{16}^{2}\to e_{16}^{10}\to e_{16}^{13}\to e_{16}^{15}\to e_{16}^{8}$ circle. The second phase is characterized by the periodical circle.

To verify that our predictions on P53 pathway progression, we examined the experimental results published on P53 pathways. The published results confirmed that 1) P53 pathway has a stable pattern pulses generation [17], and 2) there exists two-phase transition in P53 pathways [18].

## Discussion and conclusions

Reachability of Boolean networks is a central issue for network analysis. However, due to the lacking of a systemic approach to present network progression with respect to the structure and functions of a network, little is known about reachability of a complex network. Recent results are acquired with computational estimates and on structural property [8, 9, 19]. The most significant contributions of this study were listed below. We have developed a tool to determine the reachability for Boolean networks with arbitrary number of nodes and Boolean functions. This tool allows general non-engineer users to verify whether a Boolean network is reachable or not. Further, with a given Boolean network, we can recognize all the reachable states and separate them from non-reachable states. If a desired state of the network is among the reachable states, a modification of initial states through gene knock out or dosage injection may lead to desired response. Otherwise, a more complicated control should be introduced.

We also found six simplest forms for reachable 2-D boolean networks. This result provided the structure of reachable transition matrix and allowed us to examine possible modification of structure and function of a network. Finally, we proposed the first state feedback control design strategy of N-node Boolean networks. The control is determined by status of all nodes in the network and is feasible for realtime application. For instance, a possible control design was introduced to a 2-node network to form a 3-node reachable Boolean network shown in Fig. 3. Though the last Boolean function may be complicated, it provides possible direction for state feedback control design. Simplification and optimization of the state feedback control design and output feedback control design will be conducted as our future research.

Finally, we presented the analysis of the major switch in P53 pathway to predict the progression of the pathway and validated our prediction with published results.

## Methods

**Semi-tensor product.** Semi-tensor product $'\ltimes '$ allows us to multiply two matrices without the requirement of matching their dimensions [10].

For a logical dynamics, we know that ^′^1^′^ and ^′^0^′^ are used to represent logical states ^′^*True*^′^ and ^′^*False*^′^, respectively. In order to define the logical values for computing and analysis, vector forms of Boolean variables are applied using semi-tensor product paper. The semi-tensor product of two matrices $A\in \mathbb {R}^{m\times n}$ and $B\in \mathbb {R}^{p\times q}$ is that

4 $$\begin{array}{@{}rcl@{}} A\ltimes B=(A\otimes I_{\alpha/n})(B\otimes I_{\alpha/p}), \end{array} $$

where *α*=*lcm*(*n*,*p*), *lcm*(*n*,*p*) denotes the least multiple of *n* and *p*. *I*_*α*/*n*_ and *I*_*α*/*p*_ are the (*α*/*n*×*α*/*n*) identity matrix and (*α*/*p*×*α*/*p*) identity matrix, respectively. Operation ⊗ means the Kronecker product [20].

**Representation of Boolean network dynamicss using semi-tensor product.** We summarize the mathematical tool of semi-tensor product in Cheng’s papers as follows. [14, 21]

- **Cheng’s result 1:** Any logical function *f*(*x*_1_,*x*_2_,⋯,*x*_*n*_) with logical states $x_{1}, x_{2},\cdots, x_{n}\in \mathcal {D}$ can be expressed in a multi-linear form as
  5 $$ f(x_{1},x_{2},\cdots,x_{n})=M\ltimes x_{1}\ltimes x_{2} \cdots\ltimes x_{n}  $$
  where *M* is a 2×2^*n*^ logical matrix.
- **Cheng’s result 2:** Consider a Boolean network with states $x_{i}\in \mathcal {D}$ and denote integrated state $x(k)=x_{1}(k)\ltimes x_{2}(k)\cdots \ltimes x_{n}(k)$, there exists a unique matrix $L\in \{0,1\}^{2^{n}\times 2^{n}}$ such that
  6 $$ x(k+1)=L\ltimes x(k),  $$
  *L* is the transition matrix of this Boolean network.

Cheng’s results allow us to represent the dynamics of Boolean networks with an algebraic state space representation. Then, the time transition diagram can be determined by this transition matrix *L*.

**Examining all the 2-node reachable Boolean networks with logical operations.** For any 2-node (node *x*_1_ and node *x*_2_) Boolean network, denote the integrated state $x(k)=x_{1}(k)\ltimes x_{2}(k)$ as a 4×1 vector, the dynamics can be represented by a multi-linear form as below

$$ \begin{aligned} x_{1}(k+1)&=M_{1}\ltimes x_{1}(k)\ltimes x_{2}(k)\\ &=M_{1}\ltimes x(k),\\ x_{2}(k+1)&=M_{2}\ltimes x_{1}(k)\ltimes x_{2}(k)\\ &=M_{2}\ltimes x(k), \end{aligned}  $$

where *M*_1_ and *M*_2_ are the 2×4 undetermined logical matrices. Suppose

7 $$\begin{array}{@{}rcl@{}} M_{1}\,=\,\left(\begin{array}{cccc} \alpha_{11}&\alpha_{12}&\alpha_{13}&\alpha_{14} \\ \alpha_{21}&\alpha_{22}&\alpha_{23}&\alpha_{24} \end{array} \right)\!, M_{2}\,=\,\left(\begin{array}{cccc} \beta_{11}&\beta_{12}&\beta_{13}&\beta_{14} \\ \beta_{21}&\beta_{22}&\beta_{23}&\beta_{24} \end{array} \right), \end{array} $$

noticing that if one of *α*_1*j*_ and *α*_2*j*_, *j*=1,2,3,4, is zero, the other one must be one. Also, the same condition is on the situation of *β*_1*j*_ and *β*_2*j*_, *j*=1,2,3,4. In other words, *α*_1*j*_+*α*_2*j*_=1 and *β*_1*j*_+*β*_2*j*_=1, where $\alpha _{1j},\alpha _{2j}, \beta _{1j},\beta _{2j}\in \mathcal {D}$. Therefore, *M*_1_ and *M*_2_ has 2^4^ combination cases.

There exists a unique matrix *L* such that

$$x(k+1)=L\ltimes x(k), $$

where $L=M_{1}\ltimes (I_{4}\otimes M_{2})\ltimes \Phi _{2}$. Moreover, *Φ*_2_ is a fixed 16×4 matrix provided below, which only depends on the number of nodes.

8 $$\begin{array}{@{}rcl@{}} \Phi_{2}=\left(\begin{array}{cccc} 1&0&0&0 \\ 0&0&0&0\\ 0&0&0&0\\ 0&0&0&0\\ 0&0&0&0\\ 0&1&0&0\\ 0&0&0&0\\ 0&0&0&0\\ 0&0&0&0\\ 0&0&0&0\\ 0&0&1&0\\ 0&0&0&0\\ 0&0&0&0\\ 0&0&0&0\\ 0&0&0&0\\ 0&0&0&1 \end{array} \right) \end{array} $$

By substituting the expressions of *M*_1_, *M*_2_, and *Φ*_2_ into the calculation formula of *L*, the matrix *L* is obtained as

9 $$\begin{array}{@{}rcl@{}} L&=&M_{1}\ltimes (I_{4}\otimes M_{2})\ltimes \Phi_{2} \\ &=&\left(\begin{array}{cccc} \alpha_{11}\beta_{11}&\alpha_{12}\beta_{12}&\alpha_{13}\beta_{13}&\alpha_{14}\beta_{14} \\ \alpha_{11}\beta_{21}&\alpha_{12}\beta_{22}&\alpha_{13}\beta_{23}&\alpha_{14}\beta_{24}\\ \alpha_{21}\beta_{11}&\alpha_{22}\beta_{12}&\alpha_{23}\beta_{13}&\alpha_{24}\beta_{14} \\ \alpha_{21}\beta_{21}&\alpha_{22}\beta_{22}&\alpha_{23}\beta_{23}&\alpha_{24}\beta_{24} \end{array} \right). \end{array} $$

Based on Finding 1, in order to ensure it is a reachable Boolean network, the transition matrix should satisfy the *j*_*th*_ column is not $e_{2^{n}}^{j}$ and if *j*_*th*_ column is $e_{2^{n}}^{k}$, then *k*_*th*_ column is not $e_{2^{n}}^{j}$, which means transion matrix *L* here must be a special asymmetric permutation matrix, and satisfy that all the elements on the diagonal are zeros. So, there are only six different forms of *L* in total as shown below.

10 $$\begin{array}{@{}rcl@{}} L_{1}=\left(\begin{array}{cccc} 0&1&0&0 \\ 0&0&0&1\\ 1&0&0&0\\ 0&0&1&0 \end{array} \right),~~~~~~~~~~~L_{2}=\left(\begin{array}{cccc} 0&0&1&0 \\ 1&0&0&0\\ 0&0&0&1\\ 0&1&0&0 \end{array} \right) \end{array} $$

11 $$\begin{array}{@{}rcl@{}} L_{3}=\left(\begin{array}{cccc} 0&1&0&0 \\ 0&0&1&0\\ 0&0&0&1\\ 1&0&0&0 \end{array} \right),~~~~~~~~~~~L_{4}=\left(\begin{array}{cccc} 0&0&1&0 \\ 0&0&0&1\\ 0&1&0&0\\ 1&0&0&0 \end{array} \right) \end{array} $$

12 $$\begin{array}{@{}rcl@{}} L_{5}=\left(\begin{array}{cccc} 0&0&0&1 \\ 1&0&0&0\\ 0&1&0&0\\ 0&0&1&0 \end{array} \right),~~~~~~~~~~~L_{6}=\left(\begin{array}{cccc} 0&0&0&1\\ 0&0&1&0\\ 1&0&0&0\\ 0&1&0&0 \end{array} \right) \end{array} $$

According to the forms of *L*_*i*_, *i*=1,2,⋯,6, the related *M*_1_ and *M*_2_ can be determined and their corresponding most simplistic logic equations can be obtained respectively, which are listed below.

In terms of *L*_1_, *M*_1_ and *M*_2_ can be reduced as

13 $$\begin{array}{@{}rcl@{}} M_{1}=\left(\begin{array}{cccc} 0&1&0&1 \\ 1&0&1&0 \end{array} \right),~~M_{2}=\left(\begin{array}{cccc} 1&1&0&0 \\ 0&0&1&1 \end{array} \right). \end{array} $$

Then, the most simplistic Boolean network dynamics equation related to *L*_1_ is

14 $$ \begin{aligned} x_{1}(k+1)=&\neg x_{2}(k)\\ x_{2}(k+1)=&x_{1}(k). \end{aligned}  $$

All the other five sets of *M*_1_ and *M*_2_ corresponding *L*_*i*_, *i*=2,3,4,5,6, can be obtained through the same way. All possible Boolean networks and corresponding transition matrices were shown in Fig. 2.

**Extending N-node reachable network dynamics from 2-node reachable Boolean networks.** Denote *M*_*i*_, *i*=1,2,⋯,*n* as 2×2^*n*^ logical matrices of each logical function of a N-node Boolean network. Extension of the first *n* logical functions to extended *n*+1 nodes Boolean functions leads to $M^{*}_{i}$, *i*=1,2,⋯,*n*+1. Specifically, $M^{*}_{n+1}$ indicates the (2×2^*n*+1^) logical matrix of the last logical function. Moreover, the relationship between *M*_*i*_ and $M^{*}_{i}$ is

15 $$ M^{*}_{i}=E\ltimes M_{i}, i=1,2,\cdots,n,  $$

where matrix *E* is a fixed 2×4 matrix shown as

16 $$\begin{array}{@{}rcl@{}} E=\left(\begin{array}{cccc} 1&1&0&0 \\ 0&0&1&1 \end{array} \right). \end{array} $$

In terms of *n* nodes reachable Boolean network system, the corresponding expression of transition matrix *L*_*n*_ is

17 $$ L_{n}=M_{1}\ltimes \prod\limits_{i=2}^{n}[(I_{2^{n}}\otimes M_{i})\ltimes \Phi_{n}],  $$

where *M*_*i*_ are 2×2^*n*^ logical matrices of dynamics, *i*=1,2,⋯,*n*. *Φ*_*n*_ is a fixed 2^2*n*^×2^*n*^ matrix, which only depends on the number of nodes.

Then, for *n*+1 nodes reachable Boolean network system, the corresponding expression of transition matrix *L*_*n*+1_ is

$$ {{\begin{aligned} {}L_{n+1}\!=&M^{*}_{1}\ltimes \prod\limits_{i=2}^{n+1}[(I_{2^{n+1}}\otimes M^{*}_{i})\ltimes \Phi_{n+1}]\\ =&M^{*}_{1}\ltimes \prod\limits_{i=2}^{n}[(I_{2^{n+1}}\otimes M^{*}_{i})\ltimes \Phi_{n+1}]\!\ltimes((I_{2^{n+1}}\otimes M^{*}_{n+1})\ltimes \Phi_{n+1})\\ =&E\ltimes M_{1}\!\ltimes\!\! \prod\limits^{n}_{i=2}\![\!(I_{2^{n+1}}\!\otimes\! (E\!\ltimes\! M_{i}))\!\ltimes\! \!\Phi_{n+1}]\!\ltimes\!(I_{2^{n+1}}\!\!\otimes\! M^{*}_{n+1})\!\!\ltimes\!\! \Phi_{n+1}\\ =&L^{*}_{n}\ltimes\!(I_{2^{n+1}}\otimes M^{*}_{n+1})\ltimes \Phi_{n+1}, \end{aligned}}}  $$

where $L^{*}_{n}=E\ltimes M_{1}\ltimes \prod ^{n}_{i=2}[(I_{2^{n+1}}\otimes (E\ltimes M_{i}))\ltimes \Phi _{n+1}]$, which is a 2^*n*^×2^*n*+1^ matrix. Due to $(I_{2^{n+1}}\otimes M^{*}_{n+1})\ltimes \Phi _{n+1}$ is a 2^*n*+2^×2^*n*+1^ matrix, $L^{*}_{n}$ matrix will be extended as $L^{*}_{n}\otimes I_{2}$ when doing the semi-product. Therefore, *L*_*n*+1_ is a 2^*n*+1^×2^*n*+1^ matrix, which has multi-level-nested structure based on matrices *L*_2_, *L*_3_, ⋯, *L*_*n*_ of 2−*D*, 3−*D*, ⋯, *n*−*D* reachable or reachable Boolean network systems. According to the property of transition matrix, when extending to more nodes reachable networks, each 1-block will be extended to a 2×2 identity matrix or skew-identity matrix, which means a 1-block can be extended to two 1-blocks. If there is an odd number of identity matrices or skew-identity matrices, the extended Boolean network is reachable. Based on this rule, the logical matrix $M^{*}_{n+1}$ can be derived, then the feedback controller can be determined.

Starting from the six forms of 2-node reachable logical Boolean network, we can find all the 3-node corresponding reachable Boolean network dynamics with logical operations. By that analogy, N-node (*n*>2) reachable logical Boolean network can be obtained.
